# Pulmonary Hypertension Phenotypes in Systemic Sclerosis: The Right Diagnosis for the Right Treatment

**DOI:** 10.3390/ijms21124430

**Published:** 2020-06-22

**Authors:** Umberto Attanasio, Alessandra Cuomo, Flora Pirozzi, Stefania Loffredo, Pasquale Abete, Mario Petretta, Gianni Marone, Domenico Bonaduce, Amato De Paulis, Francesca Wanda Rossi, Carlo Gabriele Tocchetti, Valentina Mercurio

**Affiliations:** 1 Department of Translational Medical Sciences. Federico II University, 80131 Naples, Italy; umberto.attanasio@yahoo.it (U.A.); alebcuomo@gmail.com (A.C.); flora.pirozzi@unina.it (F.P.); stefanialoffredo@hotmail.com (S.L.); p.abete@unina.it (P.A.); petretta@unina.it (M.P.); marone@unina.it (G.M.); bonaduce@unina.it (D.B.); amato.depaulis@unina.it (A.D.P.); francescawanda.rossi@unina.it (F.W.R.); carlogabriele.tocchetti@unina.it (C.G.T.); 2 Center for Basic and Clinical Immunology Research (CISI), 80131 Naples, Italy; 3 World Allergy Organization (WAO), Center of Excellence, 80131 Naples, Italy

**Keywords:** systemic sclerosis, pulmonary hypertension, pulmonary vascular disease, pulmonary vasodilators, risk stratification

## Abstract

Systemic sclerosis is an auto-immune disease characterized by skin involvement that often affects multiple organ systems. Pulmonary hypertension is a common finding that can significantly impact prognosis. Molecular pathophysiological mechanisms underlying pulmonary hypertension in systemic sclerosis can be extremely heterogeneous, leading to distinct clinical phenotypes. In addition, different causes of pulmonary hypertension may overlap within the same patient. Since pulmonary hypertension treatment is very different for each phenotype, it is fundamental to perform an adequate diagnostic work-up to properly and promptly identify the prevalent mechanism underlying pulmonary hypertension in order to start the right therapies. When pulmonary hypertension is caused by a primary vasculopathy of the small pulmonary arteries, treatment with pulmonary vasodilators, often in an initial double-combination regimen, is indicated, aimed at reducing the mortality risk profile. In this review, we describe the different clinical phenotypes of pulmonary hypertension in the scleroderma population and discuss the utility of clinical tools to identify the presence of pulmonary vascular disease. Furthermore, we focus on systemic sclerosis-associated pulmonary arterial hypertension, highlighting the advances in the knowledge of right ventricular dysfunction in this setting and the latest updates in terms of treatment with pulmonary vasodilator drugs.

## 1. Introduction

Systemic sclerosis (SSc), also known as scleroderma, is an immune-mediated disease of the connective tissue, mainly characterized by thickening and fibrosis of the skin and internal organs [1]. Among the numerous organs and systems that can be involved, including heart, lungs, kidneys, gastro-intestinal tract and skeletal muscle system, the pulmonary circulation can also be affected, in the form of pulmonary hypertension (PH) [2]. In particular, PH is characterized by a chronic and progressive increase in the pressure in the pulmonary vascular system, which ultimately leads to right ventricular dysfunction and right-sided heart failure and can be caused by different pathophysiological mechanisms [3]. In the context of SSc, PH etiology can be extremely heterogeneous, developing as a complication of both heart or lung diseases, which are common comorbidities in scleroderma patients, or as a consequence of chronic thromboembolism. Furthermore, PH can be ascribed to a primary arteriolar vasculopathy, namely pulmonary arterial hypertension (PAH) [4]. PH etiology in SSc patients can differ substantially not only among patients, but within the same patient during scleroderma natural history. Notably, the presence of PH, beside its specific etiology, negatively affects the already impaired prognosis of scleroderma patients [5]. PAH in SSc is among the most frequent pulmonary vascular complication in SSc and its presence significantly worsens scleroderma patients’ prognosis [6]. Treatment of PAH consists of the administration of pulmonary vasodilator drugs, which target different molecular pathways involved in the balance of vasodilation and vasoconstriction mechanisms that regulate pulmonary vascular tone [3]. The aims of this review are to describe the different molecular and clinical phenotypes of PH that can affect the scleroderma population and to discuss the utility of clinical and instrumental tools to identify the presence of pulmonary vascular disease. Furthermore, we focused on SSc–PAH, highlighting the advances in the knowledge of right ventricular dysfunction in this setting and the latest updates in terms of treatment with pulmonary vasodilator drugs.

## 2. Systemic Sclerosis and Pulmonary Hypertension: A Dangerous Liaison 

SSc is a rare disorder, with an annual prevalence of one to five cases/1000 individuals, a female predilection that occurs in women four times more frequently than in males, which usually manifests around the fourth or fifth decade of life. Based on the extension of skin involvement, SSc can be classified into limited cutaneous SSc (lcSSc) and diffuse cutaneous SSc (dcSSc), with skin thickening of the limbs distal to the elbows or knees with or without face and neck involvement in lcSSc, and skin thickening of the proximal limb or trunk skin in dcSSc. Moreover, the presence of criteria for SSc, but without skin involvement, corresponds to the definition of SSc sine scleroderma [7]. Although this classification is merely based on the extension of skin involvement, there are several clinical differences, as well as specific serum autoantibody profiles, between dcSSc and lcSSc [8]. For instance, organ involvement is not only more frequent in the dcSSc, but also appears earlier in comparison to patients with lcSSc. However, organ involvement is neither exclusive nor pathognomonic of dcSSc, as it can also be present in lcSSc patients. Among the possible affected organs and systems, which include the lungs, heart, gastro-intestinal tract, kidneys, muscles and joints [8,9], pulmonary vascular system, is one of the most frequently involved, and the development of PH represents a pivotal adverse determinant of SSc patients’ prognosis. 

PH is a hemodynamic and pathophysiologic condition defined by the presence of a mean pulmonary arterial pressure (mPAP) ≥ 25 mmHg evaluated by means of resting right heart catheterization (RHC) [3]. This definition has been recently updated during the sixth World Symposium on Pulmonary Hypertension held in Nice in 2018, and it has been proposed to lower the cut-off for the diagnosis of PH to a mPAP >20 mmHg, [10]. PH can be classified as pre- and post-capillary. For instance, pre-capillary PH is characterized by a pulmonary artery wedge pressure (PAWP) ≤ 15 mmHg and, according to the new definition from Nice 2018 [10], a pulmonary vascular resistance (PVR) ≥ 3 WU, while post-capillary PH is defined by a PAWP > 15 mmHg with normal PVR [3]. The increase in afterload due to chronic elevation of pressure in the pulmonary circulation can progressively affect the right ventricle (RV), leading to RV disfunction, with the development of right-sided heart failure which can ultimately affect prognosis [11]. Based on these clinical characteristics and pathophysiological mechanisms, five different groups have been identified: Group 1 (PAH), Group 2 (PH due to left heart disease), Group 3 (PH due to lung disease or hypoxia), Group 4 (chronic thromboembolic pulmonary hypertension (CTEPH)), and Group 5 (PH due to unclear or multifactorial mechanisms). From a hemodynamic standpoint, patients in Group 1, Group 3 and Group 4 present a pre-capillary form of PH, while those in Group 2 may present both an isolated post-capillary PH or a combined PH with a pre-capillary component, given by an elevated diastolic pressure gradient or an increase in pulmonary vascular resistance [3]. 

In SSc patients, PH can be caused by different pathophysiological mechanisms and therefore can manifest with a wide spectrum of different phenotypes, ranging from primary vasculopathy of the small pulmonary arteries (Group 1 PAH), to PH due to interstitial lung disease (Group 3), and PH due to left ventricular systolic or diastolic dysfunction (Group 2) [2]. 

**Figure 1:**
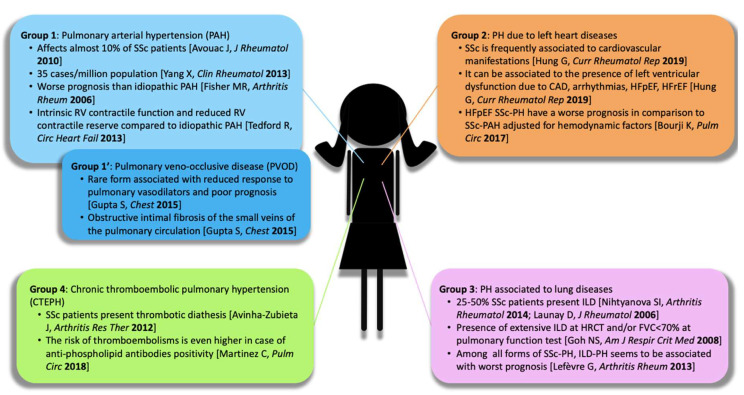
Different phenotypes of pulmonary hypertension in patients with systemic sclerosis. Abbreviations: PAH: pulmonary arterial hypertension; SSc, systemic sclerosis; RV, right ventricle; PVOD, pulmonary veno-occlusive disease; PH, pulmonary hypertension; CAD, coronary artery disease; HFpEF, Heart failure with preserved ejection fraction; HFrEF, heart failure with reduced ejection fraction; CTEPH, chronic thromboembolic pulmonary hypertension; ILD, interstitial lung disease; HRCT, high resolution computed tomography; FVC, forced vital capacity.

A careful phenotyping of PH in SSc needs to be properly and promptly addressed, in order to provide the most appropriate treatment, which is very different for each specific underlying condition [3]. Furthermore, a possible overlap of different forms of PH can occur within the same SSc patient. As stated above, PH etiology in SSc could be related to almost any of the clinical PH groups. For instance, PH can be caused by left heart dysfunction, which is a fairly common finding in SSc patients [12,13], since SSc can affect the heart as a whole (including pericardium, myocardium and, less commonly, cardiac valves) and only clinically manifests in approximately 10–30% of cases [9]. Another common cause of PH in SSc is represented by interstitial lung disease (ILD), the prevalence of which ranges between 25% and 50% [5,14]. In particular, pre-capillary PH is classified as belonging to Group 3 in the presence of the extensive ILD involvement evaluated through high-resolution computed tomography (HRCT), with a reduction in the forced vital capacity (FVC) <70% at pulmonary function test [15]. Potentially, SSc patients may develop also Group 4 as a consequence of the increased risk of pulmonary thromboembolic disease, which can be even higher when anti-phospholipid antibodies are present [16,17]. Notably, SSc patients are at risk of developing pulmonary veno-occlusive disease (PVOD), a rare and yet underdiagnosed form of PH classified as Group 1 and characterized by the obstructive intimal fibrosis of the small veins and venules of the pulmonary circle, a poor response to pulmonary vasodilators, and an extremely poor prognosis [18]. Furthermore, PH in SSc may also be caused by a primary vasculopathy and the remodeling of the small- and medium-sized pulmonary arteries, resulting in vasoconstriction, fibrosis, intraluminal micro-thrombosis and, consequently, in a progressive increase in the PVR, therefore configuring a Group 1 PAH subtype (SSc–PAH) [19]. Figure 1 summarizes the different possible phenotypes of PH in SSc patients.

From an epidemiological standpoint, a metanalysis of five European studies published in 2010 showed that, in a cohort of 1165 SSc patients, 83 had PH, with a global prevalence of 7%. The distribution of those patients showed that 77% had a pre-capillary PH, where almost 2/3 belonged to Group 1 as PAH (51% of total patients) and 1/3 belonged to Group 3 as PH secondary to ILD (26% of total patients); while 21% had post-capillary PH due to left-sided heart disease and 2% had PH due to PVOD. The prevalence of CTEPH was not assessed [20]. The Evidence-based detection of pulmonary arterial hypertension in systemic sclerosis (DETECT) study shows a similar proportion between SSc–PAH (60%) and other types of PH in SSc (40%) [21]. Furthermore, a pooled analysis conducted by Yang and colleagues in 2013, by examining 20 studies (17 on connective tissue disease associated PAH and three on idiopathic PAH) found that the prevalence of PAH in SSc patients was 35 per million population (95% CI, from 13 to 65), while the prevalence of idiopathic PAH was 12 cases per million population (95% CI 5-22 cases per million), demonstrating that, beyond the rarity of both SSc and PAH, PAH is a “common comorbidity” in SSc patients [22].

Notably, those data are based on the diagnosis of PH by the measurement of mPAP >25 mmHg, as the studies included were published before the sixth World Symposium on Pulmonary Hypertension held in 2018, so global prevalence could be higher, and the distribution of patients may vary slightly. Nevertheless, it seems that the true impact of this new definition may be minimal in terms of an increase in the number of SSc patients diagnosed with PH, because of the unalerted thresholds of PVR and PAWP working as buffer zones against its overdiagnosis, as it was recently shown that, in the DETECT study cohort, only 36 (15% of total) patients out of 244 presented a borderline mPAP (between 20 and 24 mmHg), of which then only four (1.6% of total) were classified as SSc–PAH [23].

Overall, the presence of PH, in spite of the specific etiology, significantly affects the prognosis in SSc patients. Indeed, data from the Assessing the Spectrum of Pulmonary Hypertension Identified at a REferral Centre (ASPIRE) registry showed that PH is a disease with a very poor prognosis, which may range from a 3-year survival rate of 44% for lung-correlated Group 3 PH to 73% for left-sided heart disease-correlated Group 2 PH. Notably, SSc–PAH showed a very poor prognosis, mostly related to the early development of right-sided heart failure as cause of death [24]. A metanalysis showed that, among SSc patients with PH, the global 3-year survival rate was 52%, with worse outcomes in Group 3 ILD-related PH (with a 35% 3-year survival rate) in comparison to Group 1 SSc–PAH (with a 56% 3-year survival rate) [25]. Furthermore, SSc patients with Group 2 PH due to heart failure with preserved ejection fractions showed a worse prognosis in comparison to SSc–PAH when adjusted for hemodynamic factors [12].

## 3. Pulmonary Hypertension in Systemic Sclerosis: Challenges in Screening and Diagnosis

Given the increased risk of developing PH in the SSc population and its strong impact on the mortality of those patients, it is recommended to refer patents for early and periodical screening [26,27]. One of the best and most immediate tools useful for this purpose is transthoracic echocardiography (TTE), which can estimate the probability of PH, by means of peak tricuspid regurgitation velocity measurement and evaluation of signs of increased afterload (right heart dimensions, pulmonary arterial enlargement, vena cava diameter and collapsibility) [3]. Furthermore, TTE gives important information on both right- and left-sided heart function and morphology, which can help in highlighting the possible presence of left heart disease as a cause of PH. Other important tests that should always be performed with screening purpose in all SSc patients include pulmonary function testing (PFT), including spirometry, lung volumes and diffusion capacity carbon monoxide (DLCO), which are useful to assess lung involvement early and screen for both PH and ILD [26]. TTE and PFT with DLCO can be repeated if new signs or symptoms develop or can be done annually as screening tests [26].

Once the suspect of PH is addressed, it is crucial to further characterize the patient using other key imaging tools in order to explore the possible presence of other causes of PH. HRCT is extremely useful in identification and o staging ILD as a cause of Group 3 PH [15,28], and also Group 1 PVOD [29]. Information from HRCT needs to be integrated with PFT and DLCO data. Then, another diagnostic tool useful to explore the possible presence of Group 4 CTEPH, in the suspect of pulmonary thrombosis, is the ventilation/perfusion scan (V/Q scan) [30]. At this point, once the causes of PH have been identified, the gold standard for PH definitive diagnosis is still RHC, as it represents the only instrument we have to directly measure the pressures of pulmonary vascular circulation, as well as cardiac output, both of which are necessary to calculate PVR [3]. Unfortunately, once the diagnosis is done, RHC alone can only distinguish the pre- and post-capillary components, barely separating Group 2 patients from the other groups that may share similar hemodynamic assets. To further complicate the situation, it was found that a significant proportion of PAH patients might present an occult post-capillary PH, which can be unmasked after fluid challenge during RHC [31]. 

**Figure 2:**
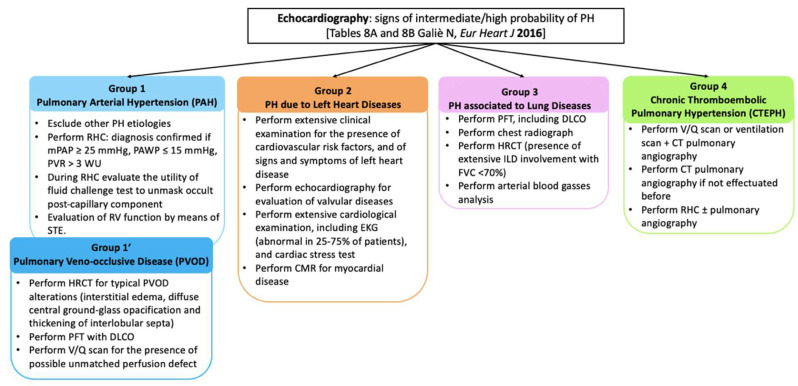
Summary of the investigation tools required to properly categorize different systemic sclerosis–pulmonary hypertension (SSc–PH) phenotypes. Abbreviations: PH: pulmonary hypertension; PAH: pulmonary arterial hypertension; RHC: right heart cathetherization; mPAP: mean pulmonary artery oressure; PAWC: pulmonary artery wedge pressure; PVR: pulmonary vascular resistance; WU: wood units; RV: right ventricle; STE: speckle-tracking echocardiography; PVOD: pulmonary veno-occlusive disease; HRCT: high-resolution computed tomogrphy; PFT: pulmonary function tests; DLCO: diffusion lung CO; V/Q: ventilation/perfusion; EKG: elettrocardiogram; CMR: cardiac magnetic resonance; ILD; interstitial lung disease; FVC: forced vital capacity; CTEPH: chronic thromboembolic pulmonary hypertension; CT computer tomography.

Remarkably, a single SSc patient can change their hemodynamic asset, switching from a phenotype to another, as Lammi and colleagues showed in a study based on the Pulmonary Hypertension Assessment and Recognition of Outcomes in Scleroderma (PHAROS) cohort: in 120 patients, they registered an overall average change in PAWP from 11 ± 5 to 13 ± 6 mmHg, so that about 30% of patients had their PAWP changed within a median time of about 1.4 years [32]. The importance of correctly phenotyping those patients is related to the need to adopt the most appropriate therapeutic strategy, and thus RHC plays a crucial role on this assessment, a single measurement alone might not be sufficient, but should be completed with serial RHC studies, clinical evaluation, other imaging data, fluid overload and/or exercise testing [33]. Figure 2 describes the utility of the available diagnostic tools according to each SSc–PH phenotype.

Once the SSc–PH phenotype has been correctly identified, a proper assessment of the mortality risk by means of a multidimensional approach is needed. This has been widely investigated in SSc–PAH patients, as described in detail afterwards in this review. The main characteristics of the disease considered for prognostication, in accordance with the PH risk stratification table from the European guidelines [3], are some clinical aspects, including syncope, symptoms, signs of RV failure and World Health Organization (WHO) functional class, exercise functional aspects, measured using the 6-minute walking distance (6MWD) and/or cardiopulmonary exercise testing (CPET) and right heart functional aspects, measured using echocardiography, cardiac magnetic resonance (CMR) imaging or RHC, and measuring the plasma levels of N-Terminal Pro-Brain Natriuretic Peptide (NT-proBNP) [3]. Interestingly, the measurement of NT-proBNP may suggest the presence of PAH with an elevated sensitivity and specificity in SSc patients when its serum levels are elevated by more than two times the reference upper limit [34]. As for the exercise testing, CPET represents a non-invasive exam that can quantify the degree of relative hypoperfusion of the lungs and systemic circulation during exercise [27], thus measuring the grade of exercise limitation of the patient, which is related to patient mortality. On the other hand, the 6MWD represent a simple yet consistent tool that relates to CPET results in terms of functional impairment and patients’ mortality, and it can be considered a quicker and more affordable way of testing patients’ exercise limitations, even though it provides a smaller amount of functional data in comparison to CPET [35,36]. Since RV function strictly correlates with prognosis, especially in SSc–PAH patients [37], the evaluation of right heart functionality has to be done frequently, thus making TTE one of the most useful non-invasive tools available. Based on 2D echocardiography images, another modality of assessing cardiac motility and deformation is speckle-tracking echocardiography (STE). Originally applied for evaluating left heart function, it has been progressively tested for the right-sided heart as well. In particular, it has been also tested on SSc–PAH population with encouraging results and the advantage of adding more depth to a non-invasive assessment tool such as TTE [38]. 

Notably, to date, no multiparametric approach for the prognostication of mortality in SSc–PH has considered the impact of the specific PH etiology (i.e., the PH phenotype) on prognosis. This represents a major limitation, since, as described above, the presence of a specific PH phenotype can, in itself, significantly influence the mortality of SSc patients [12,25,39].

## 4. Molecular and Pathophysiological Features of Idiopathic PAH versus SSc–PAH: Same Vascular Disease but Different Right Ventricular Adaptation

Apart from its specific etiology, PAH is a progressive disease associated with a poor prognosis [40,41]. Interestingly, when looking into the prognoses of patients according to this specific etiology, patients with connective tissue disease-associated PAH, and especially SSc–PAH, have a significantly worse prognosis in comparison to patients with idiopathic PAH. In particular, while idiopathic PAH 1- and 3-year survival rates are estimated to be 97.4% and 88.6%, respectively, SSc–PAH patients have 1- and 3-year survival rates of 82.5% and 48.2%, respectively [6]. Notably, the prognosis is considerably worsened by the presence of symptoms and signs of right-sided heart failure [6]. Considering that scleroderma is a systemic disease that can affect different organs, including the heart, researchers have investigated whether SSc patients’ RV is further impaired, primarily by the immune disease, in comparison to idiopathic PAH patients. In 2013, Tedford and colleagues invasively analyzed not only pulmonary artery pressures and mean flow in SSc–PAH, SSc-ILD-PH and idiopathic PAH patients, but also the difference in terms of RV and pulmonary artery elastance between idiopathic PAH and SSc–PAH patients. Notably, they demonstrated that, while pulmonary artery elastance was similar in all forms of PAH, RV elastance, expressed by the slope of the end systolic pressure–volume relationship during preload reduction (Valsalva maneuver), was significantly worse in SSc–PAH patients in comparison to idiopathic PAH [37]. This suggests that one of the mechanisms underlying the worse prognosis in SSc–PAH patients might be the presence of worse intrinsic RV contractile function. Furthermore, it has been observed that a lower grade of adaptative RV hypertrophy is present in SSc–PAH compared to idiopathic PAH [42]. Moreover, Hsu and colleagues evaluated RV pressure–volume relation by means of RHC at rest and during exercise and demonstrated that SSc–PAH patients present a significantly reduced RV contractile reserve in comparison to idiopathic PAH [43]. The pathophysiology underlying the RV dysfunction in SSc–PAH seems to be related to the profibrotic processes that typically characterize scleroderma. Indeed, RV myofilaments from endocardia biopsies of patients affected by PAH have been studied, demonstrating that SSc–PAH patients present a greater extent of interstitial fibrosis compared to idiopathic PAH and SSc without PAH [44]. Furthermore, from a molecular standpoint, it has been demonstrated that sarcomeres are largely affected in SSc–PAH compared to idiopathic PAH. Indeed, RV myofilaments isolated from SSc–PAH patients have a diminished contractile force and abnormal calcium sensitivity compared with control myofilaments, in contrast to the hypercontractile compensation observed in idiopathic PAH [44]. Additionally, these findings correlate with the in vivo RV function at rest and the RV contractile reserve during exercise [44]. However, considering the invasiveness of these measurements, researchers have been focusing on finding non-invasive tools to assess RV function that could be applicable in daily clinical practice. In order to characterize the possible impairment of RV function, the role of STE as additional tool as well as conventional echocardiography in order to evaluate the elastic deformation properties of the myocardium has been explored—in particular, the longitudinal systolic deformation, in the setting of SSc–PAH. It has been demonstrated that RV longitudinal systolic strain (RVLSS) is worse in SSc–PAH patients compared to idiopathic PAH patients, with a more relevant alterations in the contractility of the basal segments of the RV free wall, but also that such RVLSS alterations correlate with prognosis, even after adjustment for gender and age, giving a useful non-invasive tool for clinicians to assess RV dysfunction and thus mortality risk in SSc–PAH patients [38]. Interestingly, treatment with pulmonary vasodilator drugs was able to significantly improve RVLSS in SSc–PAH [45].

## 5. General Considerations on Treatment of Pulmonary Hypertension in SSc

Since PH in SSc can be caused by different pathophysiologic mechanisms, each of them deserves a different therapeutic approach. In fact, the primary goal of the treatment in Group 2 PH is to optimize the underlying left heart condition according to the latest recommendations, such as heart failure, valvulopathies, arrhythmias or pericardial diseases [9], while also considering the use of diuretics to balance the fluid volume load [3]; on the other hand, Group 3 ILD-PH can be treated by addressing the lung disease, using immunosuppressive therapy and/or nintedanib [46] when indicated. A recent study demonstrated that nintedanib was able to slow the progressive decline of FVC in ILD-SSc patients [46]. Concerning the effects of immunosuppressive drugs, while a meta-analysis failed to show a significant benefit of the use of cyclofosfamide on lung function in SSc-ILD patients [47], a prospective trial demonstrated significant improvement in DLCO and a non-significant improvement in FVC with mycofenolate mofetil [48], as also confirmed in a subsequent meta-analysis [49]. Nevertheless, Group 3 PH in SSc patients still have a very poor prognosis [39]. Group 4 CTEPH, instead, in addition to life-long oral anticoagulation, needs to be approached by evaluating the possible use of chirurgical thromboendarterectomy, or, if surgery is not feasible, balloon angioplasty; otherwise, the administration of riociguat is recommended [3]. While the therapy of the former groups mainly targets the condition at the origin of PH, Group 1 PAH, being the vessels in the pulmonary circle primarily affected, is the only type of PH that can benefit from specific pulmonary vasodilator drugs. These drugs target the vascular pathways that are impaired in PAH, and act against pulmonary arterial remodeling and chronic vasoconstriction [3,50]. Unfortunately, studies on the use of specific pulmonary vasodilator drugs for the treatment of PH, other than for Group 1, have shown that such a treatment is ineffective [51,52,53] or even harmful [54]. Therefore, aside from Group 1 PAH, there is no evidence on using pulmonary vasodilators in PH due to other etiologies.

## 6. Molecular Pathways Targeted by PAH-Specific Therapies 

The pulmonary vascular endothelial dysfunction that typically characterizes patients with PAH is the result of an imbalance between endogenous vasoconstrictors and proliferative promoters, i.e., entothelin-1, which are upregulated, and vasodilatory mediators, i.e., nitric oxide (NO) and prostacyclin, which are decreased in production. Indeed, endothelin-1, NO and prostacyclin pathways are the major molecular pathways involved in the pathogenesis of PAH and represent the targets of all currently approved PAH-specific therapies, as well as for SSc–PAH [50]. 

The endothelin pathway can be blocked by endothelin receptor antagonists. Ambrisentan, bosentan and macitentan are the three endothelin receptor antagonists (ERA) approved for the treatment of SSc–PAH [55]. Ambrisentan, the only endothelin-1 selective antagonist, was able to improve 6MWD in connective tissue disease (CTD)-associated PAH patients, even though survival was higher in idiopathic PAH patients, in the Ambrisentan in Pulmonary Arterial Hypertension, Randomized, Double-Blind, Placebo-Controlled, Multicenter, Efficacy Study (ARIES) 1 and 2 trials [56,57]. Bosentan was studied in the Bosentan Randomized Trial of Endothelin Antagonist Therapy (BREATHE-1) trial, where the magnitude of improvement in 6MWD of SSc–PAH patients was lower than the one of idiopathic PAH patients, but still prevented the worsening of the disease clinically [58]; moreover, it proved in the Randomized, Double-Blind, Placebo-Controlled Study with Bosentan on the Healing and Prevention of Ischemic Digital Ulcers in Patients with Systemic Sclerosis (RAPIDS-2) study to prevent the formation of digital ulcers, but not their healing rate [59]. Finally, macitentan, a newer molecule of this class of drugs, demonstrated a safer hepatotoxicity profile [60] and proved to reduce the mortality of PAH patients in the Study with an Endothelin Receptor Antagonist in Pulmonary Arterial Hypertension to Improve Clinical Outcome (SERAPHIN) trial, with similar results between CTD–PAH and idiopathic PAH patients [61,62]. A further analysis on the use of macitentan in SSc–PAH was offered from the OPsumit USers (OPUS) prospective registry, which characterized the use and safety profile of this drug in a real-world setting, where patients who has recently started a therapy with macitentan were eligible for enrollment in this registry. The study showed a similar safety profile and survival between the 191 SSc–PAH and the 577 idiopathic PAH patients [63]. Data on the use of macitentan in the real-world setting were described in the OPsumit Historical USers cohort (OrPHeUS) retrospective study, in which were included PAH patients who recently started taking macitentan. The data analysis showed similar clinical outcomes (also in terms of survival) and safety profiles between the 659 SSc–PAH and the 2283 idiopathic and hereditary PAH patients [64].

Concerning the NO pathway, the reduction in levels of cGMP, the mediator of NO-dependent smooth muscle cell relaxation, can be antagonized by two different pharmacological approaches: the activation of soluble guanylate cyclase (sGC) with the sGC stimulator riociguat or the inhibition of the degradation of cGMP mediated by phosphodiesterase-5 (PDE-5) with sildenafil or tadalafil [50]. Those two classes of drugs cannot be concomitantly administered because of the increase in adverse effects, as demonstrated in the Pulmonary Arterial Hypertension Soluble Guanylate Cyclase-Stimulator Plus Trial (PATENT) [65]. The efficacy of sildenafil and tadalafil had a proven efficacy in clinical trials that included CTD–PAH patients, which resulted in sildenafil having a similar efficacy in CTD–PAH and idiopathic PAH patients in improving the 6MWD [66,67] and tadalafil having a slightly lower efficacy when comparing the same two classes of patients [68,69]. Notably, both demonstrated an effect in preventing or healing the digital ulcers in patients with SSc [70]. Riociguat was first evaluated in a 12-week trial with 443 symptomatic PAH patients (66 of which were SSc–PAH) called PATENT-1 [71] and then in an extension study of the same trial, called PATENT-2 [72], where it proved to consistently improve 6MWD and WHO functional class for up one year even in PAH-CTD patients, where those improvements persisted at 2 years with a similar 2-year survival to idiopathic PAH patients [73].

Finally, prostacyclin vasodilation, mediated by the stimulation of prostaglandin-I2 receptor (IP), can be enhanced by two classes of drugs that can activate this receptor: the selective IP-receptor agonist Prostaglandin-I2 (PGI2) analogue (selexipag), which can be orally administered and the non-prostanoid IP-receptor agonists (epoprostenol, treprostinil and iloprost) [51]. Selexipag was studied in the PGI2 Receptor Agonist In Pulmonary Arterial Hypertension (GRIPHON) trial [74], where it proved to reduce patients’ risk of death or complications related to PAH and a CTD–PAH sub-analysis confirmed those results for the 170 SSc–PAH patients involved in the trial, even though those patients were more impaired at baseline [75]. Despite the uncomfortable method of administration of non-prostanoid IP agonists, their efficacy has been proven for PAH and, in particular, for SSc–PAH patients as well [76,77,78], though this class of drugs is usually recommended for patients in WHO functional class III or IV or in intermediate–high risk classes [3].

## 7. Risk Stratification, Goals of PAH-specific Therapy, and Combination Therapy

Considering that PAH severely impacts both quality and expectancy of life, patients need to be promptly evaluated for 1-year mortality risk and stratified accordingly, in order to identify the most adequate treatment strategy [79]. Notably, thanks to the numerous clinical registries created over the past decades, it was possible to validate different scores, mostly based on European PH guidelines from 2015, which aim to assess the 1-year mortality risk of PAH patients [3]. In particular, the European 2015 PH guidelines present a multidimensional approach for risk stratification with 13 variables and nine items, divided into three distinct domains, exploring clinical status, exercise capacity and right-sided heart function of the patient, assessed by clinical evaluation, serum biomarkers, RHC, echocardiography or CMR, and 6MWD. Each variable present specific cut-offs for low-, intermediate- or high-risk, which are also referred as “green”, “yellow” or “red” criteria respectively [3]. Recently, data from the Registry to Evaluate Early and Long-Term PAH Disease Management (REVEAL) [80], the French PH Network (FPHN) [81], the Swedish PAH Registry (SPAHR) [82] and the Prospective Registry of Newly Initiated Therapies for Pulmonary Hypertension (COMPERA) [83], were analyzed to create simplified methods that are useful to predict prognosis by combining different parameters. In particular, the latest scientific literature has focused on the prognostic role of some of the clinical, functional, and hemodynamic variables described in the European 2015 PH guidelines, specifically on 6MWD, WHO functional class, BNP or NT-proBNP, right atrial pressure (RAP), cardiac index (CI), and mixed venous oxygen saturation > 65% [3]. For instance, the FPHN method was based on counting the number of low-risk (“green”) criteria, particularly 6MWD > 440 m, WHO functional class I-II, RAP < 8mmHg, CI ≥ 2.5 L/min. Accordingly, the presence of three or four “green” criteria identifies a low-risk profile, while the presence of none of the “green” criteria, or at least one “red” criterion identifies high-risk patients. Moreover, the additional value of BNP < 50ng/L or NT-proBNP < 300 ng/L, was explored as well, confirming the prognostic validity of this approach [82]. On the other hand, the COMPERA and SPAHR methods, also called the European methods, are based on averaging the number obtained by adding one, two or three for each of the “green”, “yellow” or “red” criteria, respectively, among the seven variables described above, as presented by the patient, and dividing the sum, rounded to the nearest integer, to define the risk profile [82,83]. The only difference between the COMPERA and the SPHAR approaches is that the second one takes into account the presence or absence of pericardial effusion but does not consider CI [82]. Although a multitude of different parameters and calculators are available, most recent guidelines recommend a flexible approach to PAH risk assessment, only using modifiable variables with proved prognostic significance, in order to classify patients as low-, intermediate- or high-risk patients, depending on expected 1-year mortality. Firstly, assessing a patient’s risk at baseline is fundamental to define the correct therapeutic strategy, considering that the main goal is achieving a low-risk status, associated with an annual mortality of <5%. Moreover, both the French and European methods described above proved that follow-up risk stratification was pivotal for outcome prediction, despite baseline stratification [3]. Since SSc is not a treatable condition, the therapy of SSc–PAH aims at slowing PAH progression, achieving the lowest risk profile possible. When these risk prediction models were applied to a 151-strong SSc–PAH patient population, whether using the “French” (FPHN) method or the “European” (SPAHR and COMPERA) approach, the results confirmed the accuracy of the guideline risk table in predicting patients’ survival. Notably, only a small percentage of patients (22%) achieved a low-risk profile at follow-up [84].

The existence of three distinct pathways, as shown above, currently targeted using the available therapies, allows us to combine different drugs to achieve better results, lowering doses and (consequently) limiting the side effects of therapy, as already happens for the treatment of other clinical conditions, such as heart failure and systemic hypertension [3]. In a meta-analysis of six trials, for a total of 858 PAH patients, combination treatment was proven to reduce clinical worsening, increase the 6MWD and improve RHC results in comparison to the control group [85], while a recent analysis of the SERAPHIN and GRIPHON trials demonstrated a decrease in morbidity and mortality with the addition of, respectively, macitentan and selexipag on top of the patient’s background therapy [86,87]. Moreover, recent guidelines also highlight the importance of switching from the previous approach based on sequential combination therapy to a new method based on upfront combination treatment [3]. Furthermore, an evidence-based treatment algorithm was proposed, mainly focused on the patient’s risk class and its clinical response to initial treatment [3]. For instance, in the Ambrisentan and Tadalafil in Patients with Pulmonary Arterial Hypertension (AMBITION) trial, an initial combination treatment with ambrisentan and tadalafil was administered to 253 PAH patients, while 126 and 121 patients were administered, respectively, with ambrisentan or tadalafil as a monotherapy [88]. This was the first time that initial combination treatment was proven to have a better clinical and serological (in terms of NT-proBNP serum levels) response and a greater improvement in 6MWD in comparison to monotherapy [88]. As for the SSc–PAH population, a subgroup analysis from the AMBITION trial on 118 SSc–PAH patients confirmed the clinical benefit of upfront combination therapy [89]. Concerning upfront combination therapy with ambrisentan and tadalafil, a prospective, multicenter, open-label trial on 24 treatment-naïve SSc–PAH patients showed an improvement in hemodynamics, RV structure and function, and functional status [90], while another prospective, multicenter, open-label clinical trial on 24 SSc–PAH naïve patients highlighted a significant improvement in hemodynamics, clinical status and regional and global RV contractility (evaluated by STE echocardiography) [45].

## 8. Further Perspectives

From a pathophysiological standpoint, pulmonary vascular disease genesis intricately involves other molecular pathways besides the three already described above that are targeted by specific vasodilator drugs. Nevertheless, other molecular pathways are under investigation to find new targets that may improve PAH treatment and eventually its prognosis, which still remains extremely bleak. Among the molecules currently under investigation to be used in SSc–PAH, it is worth mentioning ifetroban, a thromboxane A2/prostaglandin H2 receptor agonist [91], dimethyl fumarate, an immunomodulator agent actually used for the treatment of psoriasis [92], bardoxolone methyl, an inducer of nuclear factor erythroid 2-related factor and a suppressor of nuclear factor kB [93] and rituximab, a monoclonal antibody against the CD20 protein of the B-cell cellular membrane, even though preliminary data on the usage of that antibody in SSc–PAH patients did not show a statistical improvement in their 6MWD [94,95]. 

## 9. Conclusions

PH represents a common and severe complication of SSc, which may present heterogeneous molecular pathophysiological mechanisms in the context of scleroderma patients, resulting in different phenotypes, with different prognoses, which require specific treatments. Nevertheless, considering that all forms of PH are characterized by high mortality rates, with a relevant impact on the already compromised survival of SSc patients, it is crucial to diagnose early, phenotype and properly treat each different form of PH in SSc patients. Notably, PH might be secondary to the lung or heart diseases that are commonly present in SSc in patients, or to chronic thromboembolism. For this reason, it is fundamental to perform a complete diagnostic work-up in SSc patients in order to identify any underlying conditions that may explain the increase in pulmonary pressure. On the other hand, SSc–PAH, which primarily affects arteriolar pulmonary circulation, represents one of the most-studied phenotypes of PH in SSc, and has an extremely poor prognosis, but can significantly benefit from specific therapy. 

The most recent recommendations highlight the importance of classifying PAH patients into low-, intermediate- or high-risk categories, according to 1-year mortality, based on clinical, functional and hemodynamic parameters. The identification of the patient’s risk profile guides treatment choices, which are aimed at achieving the lowest risk profile possible. Furthermore, guidelines recommend upfront combination treatment over mono- or sequential therapy, as recent randomized controlled clinical trials confirmed the superiority of initial combination therapy in terms of survival and/or functional parameters. 

## Abbreviations

PAH	pulmonary arterial hypertension
SSc	systemic sclerosis
RV	right ventricle
PVOD	pulmonary veno-occlusive disease
PH	pulmonary hypertension
CAD	coronary artery disease
HFpEF	heart failure with preserved ejection fraction
HFrEF	heart failure with reduced ejection fraction
CTEPH	chronic thromboembolic pulmonary hypertension
ILD	interstitial lung disease
HRCT	high-resolution computed tomography
FVC	forced vital capacity.

## References

[1] Denton C.P., Khanna D. (2017). Systemic sclerosis. Lancet.

[2] Hachulla E., De Groote P., Gressin V., Sibilia J., Diot E., Carpentier P., Mouthon L., Hatron P.Y., Jego P., Allanore Y. (2009). The three-year incidence of pulmonary arterial hypertension associated with systemic sclerosis in a multicenter nationwide longitudinal study in France. Arthritis Rheum..

[3] Galiè N., Humbert M., Vachiery J.-L., Gibbs S., Lang I., Torbicki A., Simonneau G., Peacock A., Vonk Noordegraaf A., Beghetti M. (2016). 2015 ESC/ERS Guidelines for the diagnosis and treatment of pulmonary hypertension: The Joint Task Force for the Diagnosis and Treatment of Pulmonary Hypertension of the European Society of Cardiology (ESC) and the European Respiratory Society (ERS): Endo. Eur. Heart J..

[4] Launay D., Sobanski V., Hachulla E., Humbert M. (2017). Pulmonary hypertension in systemic sclerosis: Different phenotypes. Eur. Respir. Rev..

[5] Nihtyanova S.I., Schreiber B.E., Ong V.H., Rosenberg D., Moinzadeh P., Coghlan J.G., Wells A.U., Denton C.P. (2014). Prediction of pulmonary complications and long-term survival in systemic sclerosis. Arthritis Rheumatol..

[6] Fisher M.R., Mathai S.C., Champion H.C., Girgis R.E., Housten-Harris T., Hummers L., Krishnan J.A., Wigley F., Hassoun P.M. (2006). Clinical differences between idiopathic and scleroderma-related pulmonary hypertension. Arthritis Rheum..

[7] LeRoy E.C., Medsger J. (2001). Criteria for the classification of early systemic sclerosis. J. Rheumatol..

[8] Hachulla E., Launay D. (2011). Diagnosis and classification of systemic sclerosis. Clin. Rev. Allergy Immunol..

[9] Hung G., Mercurio V., Hsu S., Mathai S.C., Shah A.A., Mukherjee M. (2019). Progress in Understanding, Diagnosing, and Managing Cardiac Complications of Systemic Sclerosis. Curr. Rheumatol. Rep..

[10] Simonneau G., Montani D., Celermajer D.S., Denton C.P., Gatzoulis M.A., Krowka M., Williams P.G., Souza R. (2019). Haemodynamic definitions and updated clinical classification of pulmonary hypertension. Eur. Respir. J..

[11] Chin K.M., Kim N.H.S., Rubin L.J. (2005). The right ventricle in pulmonary hypertension. Coron. Artery Dis..

[12] Bourji K.I., Kelemen B.W., Mathai S.C., Damico R.L., Kolb T.M., Mercurio V., Cozzi F., Tedford R.J., Hassoun P.M. (2017). Poor survival in patients with scleroderma and pulmonary hypertension due to heart failure with preserved ejection fraction. Pulm. Circ..

[13] Fernández-Codina A., Simeón-Aznar C.P., Pinal-Fernandez I., Rodríguez-Palomares J., Pizzi M.N., Hidalgo C.E., Del Castillo A.G., Prado-Galbarro F.J., Sarria-Santamera A., Fonollosa-Plà V. (2017). Cardiac involvement in systemic sclerosis: Differences between clinical subsets and influence on survival. Rheumatol. Int..

[14] Launay D., Remy-Jardin M., Michon-Pasturel U., Mastora I., Hachulla E., Lambert M., Delannoy V., Queyrel V., Duhamel A., Matran R. (2006). High resolution computed tomography in fibrosing alveolitis associated with systemic sclerosis. J. Rheumatol..

[15] Goh N.S.L., Desai S.R., Veeraraghavan S., Hansell D.M., Copley S.J., Maher T.M., Corte T.J., Sander C.R., Ratoff J., Devaraj A. (2008). Interstitial lung disease in systemic sclerosis: A simple staging system. Am. J. Respir. Crit. Care Med..

[16] Aviña-Zubieta J., Lacaille D., Sayre E., Kopec J., Choi H., Esdaile J. (2012). Risk of pulmonary embolism and deep vein thrombosis in systemic lupus erythematosus: A population-based cohort study. Arthritis Res. Ther..

[17] Martinez C., Wallenhorst C., Teal S., Cohen A.T., Peacock A.J. (2018). Incidence and risk factors of chronic thromboembolic pulmonary hypertension following venous thromboembolism, a population-based cohort study in England. Pulm. Circ..

[18] Gupta S., Gupta A., Ocak I., Domsic R., Schneider F., George P. (2015). PVOD Is Highly Prevalent in Scleroderma Patients Undergoing Lung Transplant. Chest.

[19] Chaisson N.F., Hassoun P.M. (2013). Systemic sclerosis-associated pulmonary arterial hypertension. Chest.

[20] Avouac J., Airò P., Meune C., Beretta L., Dieude P., Caramaschi P., Tiev K., Cappelli S., Diot E., Vacca A. (2010). Prevalence of pulmonary hypertension in systemic sclerosis in European Caucasians and metaanalysis of 5 studies. J. Rheumatol..

[21] Coghlan J.G., Denton C.P., Grünig E., Bonderman D., Distler O., Khanna D., Müller-Ladner U., Pope J.E., Vonk M.C., Doelberg M. (2014). Evidence-based detection of pulmonary arterial hypertension in systemic sclerosis: The DETECT study. Ann. Rheum. Dis..

[22] Yang X., Mardekian J., Sanders K.N., Mychaskiw M.A., Thomas J. (2013). Prevalence of pulmonary arterial hypertension in patients with connective tissue diseases: A systematic review of the literature. Clin. Rheumatol..

[23] Jaafar S., Visovatti S., Young A., Huang S., Cronin P., Vummidi D., McLaughlin V., Khanna D. (2019). Impact of the revised haemodynamic definition on the diagnosis of pulmonary hypertension in patients with systemic sclerosis. Eur. Respir. J..

[24] Hurdman J., Condliffe R., Elliot C.A., Davies C., Hill C., Wild J.M., Capener D., Sephton P., Hamilton N., Armstrong I.J. (2012). ASPIRE registry: Assessing the Spectrum of Pulmonary hypertension Identified at a. Eur. Respir. J..

[25] Lefèvre G., Dauchet L., Hachulla E., Montani D., Sobanski V., Lambert M., Hatron P.Y., Humbert M., Launay D. (2013). Survival and prognostic factors in systemic sclerosis-associated pulmonary hypertension: A systematic review and meta-analysis. Arthritis Rheum..

[26] Khanna D., Gladue H., Channick R., Chung L., Distler O., Furst D.E., Hachulla E., Humbert M., Langleben D., Mathai S.C. (2013). Recommendations for screening and detection of connective tissue disease-associated pulmonary arterial hypertension. Arthritis Rheum..

[27] Frost A., Badesch D., Gibbs J.S.R., Gopalan D., Khanna D., Manes A., Oudiz R., Satoh T., Torres F., Torbicki A. (2019). Diagnosis of pulmonary hypertension. Eur. Respir. J..

[28] Zompatori M., Leone M.B., Giannotta M., Galiè N., Palazzini M., Bacchi Reggiani M.L., Bono L., Pollini G.S. (2013). Ipertensione polmonare e sclerodermia: Il ruolo della TC ad alta risoluzione. Radiol. Medica.

[29] Günther S., Jaïs X., Maitre S., Bérezné A., Dorfmüller P., Seferian A., Savale L., Mercier O., Fadel E., Sitbon O. (2012). Computed tomography findings of pulmonary venoocclusive disease in scleroderma patients presenting with precapillary pulmonary hypertension. Arthritis Rheum..

[30] Galie N., Hoeper M.M., Humbert M., Torbicki A., Vachiery J.-L., Barbera J.A., Beghetti M., Corris P., Gaine S., Gibbs J.S. (2009). Guidelines for the diagnosis and treatment of pulmonary hypertension. Eur. Respir. J..

[31] Fox B.D., Shimony A., Langleben D., Hirsch A., Rudski L., Schlesinger R., Eisenberg M.J., Joyal D., Hudson M., Boutet K. (2013). High prevalence of occult left heart disease in scleroderma-pulmonary hypertension. Eur. Respir. J..

[32] Lammi M.R., Saketkoo L.A., Gordon J.K., Steen V.D. (2018). Changes in hemodynamic classification over time are common in systemic sclerosis-associated pulmonary hypertension: Insights from the PHAROS cohort. Pulm. Circ..

[33] Mercurio V., Hassoun P.M. (2018). Phenotyping pulmonary hypertension in systemic sclerosis: A moving target. Pulm. Circ..

[34] Allanore Y., Borderie D., Meune C., Cabanes L., Weber S., Ekindjian O.G., Kahan A. (2003). N-Terminal Pro-Brain Natriuretic Peptide as a Diagnostic Marker of Early Pulmonary Artery Hypertension in Patients with Systemic Sclerosis and Effects of Calcium-Channel Blockers. Arthritis Rheum..

[35] Oudiz R.J. (2005). The role of exercise testing in the management of pulmonary arterial hypertension. Semin. Respir. Crit. Care Med..

[36] de Camargo V.M., Martins B.d.C.D.S., Jardim C., Fernandes C.J.C., Hovnanian A., Souza R. (2009). Validation of a treadmill six-minute walk test protocol for the evaluation of patients with pulmonary arterial hypertension. J. Bras. Pneumol..

[37] Tedford R.J., Mudd J.O., Girgis R.E., Mathai S.C., Zaiman A.L., Housten-Harris T., Boyce D., Kelemen B.W., Bacher A.C., Shah A.A. (2013). Right ventricular dysfunction in systemic sclerosis-associated pulmonary arterial hypertension. Circ. Hear. Fail..

[38] Mukherjee M., Mercurio V., Tedford R.J., Shah A.A., Hsu S., Mullin C.J., Sato T., Damico R., Kolb T.M., Mathai S.C. (2017). Right ventricular longitudinal strain is diminished in systemic sclerosis compared with idiopathic pulmonary arterial hypertension. Eur. Respir. J..

[39] Mathai S.C., Hummers L.K., Champion H.C., Wigley F.M., Zaiman A., Hassoun P.M., Girgis R.E. (2009). Survival in pulmonary hypertension associated with the scleroderma spectrum of diseases: Impact of interstitial lung disease. Arthritis Rheum..

[40] Humbert M., Sitbon O., Chaouat A., Bertocchi M., Habib G., Gressin V., Yaïci A., Weitzenblum E., Cordier J.F., Chabot F. (2010). Survival in patients with idiopathic, familial, and anorexigen-associated pulmonary arterial hypertension in the modern management era. Circulation.

[41] Benza R.L., Gomberg-Maitland M., Elliott C.G., Farber H.W., Foreman A.J., Frost A.E., McGoon M.D., Pasta D.J., Selej M., Burger C.D. (2019). Predicting Survival in Patients With Pulmonary Arterial Hypertension: The REVEAL Risk Score Calculator 2.0 and Comparison With ESC/ERS-Based Risk Assessment Strategies. Chest.

[42] Kelemen B.W., Mathai S.C., Tedford R.J., Damico R.L., Corona-Villalobos C., Kolb T.M., Chaisson N.F., Harris T.H., Zimmerman S.L., Kamel I.R. (2015). Right ventricular remodeling in idiopathic and scleroderma-associated pulmonary arterial hypertension: Two distinct phenotypes. Pulm. Circ..

[43] Hsu S., Houston B.A., Tampakakis E., Bacher A.C., Rhodes P.S., Mathai S.C., Damico R.L., Kolb T.M., Hummers L.K., Shah A.A. (2016). Right ventricular functional reserve in pulmonary arterial hypertension. Circulation.

[44] Hsu S., Kokkonen-Simon K.M., Kirk J.A., Kolb T.M., Damico R.L., Mathai S.C., Mukherjee M., Shah A.A., Wigley F.M., Margulies K.B. (2018). Right ventricular myofilament functional differences in humans with systemic sclerosis-associated versus idiopathic pulmonary arterial hypertension. Circulation.

[45] Mercurio V., Mukherjee M., Tedford R.J., Zamanian R.T., Khair R.M., Sato T., Minai O.A., Torres F., Girgis R.E., Chin K. (2018). Improvement in right ventricular strain with ambrisentan and tadalafil upfront therapy in scleroderma-associated pulmonary arterial hypertension. Am. J. Respir. Crit. Care Med..

[46] Distler O., Highland K.B., Gahlemann M., Azuma A., Fischer A., Mayes M.D., Raghu G., Sauter W., Girard M., Alves M. (2019). Nintedanib for systemic sclerosis-associated interstitial lung disease. N. Engl. J. Med..

[47] Nannini C., West C.P., Erwin P.J., Matteson E.L. (2008). Effects of cyclophosphamide on pulmonary function in patients with scleroderma and interstitial lung disease: A systematic review and meta-analysis of randomized controlled trials and observational prospective cohort studies. Arthritis Res. Ther..

[48] Liossis S.N.C., Bounas A., Andonopoulos A.P. (2006). Mycophenolate mofetil as first-line treatment improves clinically evident early scleroderma lung disease. Rheumatology.

[49] Tzouvelekis A., Galanopoulos N., Bouros E., Kolios G., Zacharis G., Ntolios P., Koulelidis A., Oikonomou A., Bouros D. (2012). Effect and safety of mycophenolate mofetil or sodium in systemic sclerosis-associated interstitial lung disease: A meta-analysis. Pulm. Med..

[50] Humbert M., Lau E.M.T., Montani D., Jaïs X., Sitbon O., Simonneau G. (2014). Advances in therapeutic interventions for patients with pulmonary arterial hypertension. Circulation.

[51] Kolb M., Raghu G., Wells A.U., Behr J., Richeldi L., Schinzel B., Quaresma M., Stowasser S., Martinez F.J. (2018). Nintedanib plus sildenafil in patients with idiopathic pulmonary fibrosis. N. Engl. J. Med..

[52] King C.S., Shlobin O.A. (2020). The Trouble with Group 3 Pulmonary Hypertension in Interstitial Lung Disease: Dilemmas in Diagnosis and the Conundrum of Treatment. Chest.

[53] Kido K., Coons J.C. (2019). Efficacy and Safety of the Use of Pulmonary Arterial Hypertension Pharmacotherapy in Patients with Pulmonary Hypertension Secondary to Left Heart Disease: A Systematic Review. Pharmacotherapy.

[54] Vachiéry J.L., Delcroix M., Al-Hiti H., Efficace M., Hutyra M., Lack G., Papadakis K., Rubin L.J. (2018). Macitentan in pulmonary hypertension due to left ventricular dysfunction. Eur. Respir. J..

[55] Almaaitah S., Highland K.B., Tonelli A.R. (2020). Management of pulmonary arterial hypertension in patients with systemic sclerosis. Integr. Blood Press. Control..

[56] Galiè N., Olschewski H., Oudiz R.J., Torres F., Frost A., Ghofrani H.A., Badesch D.B., McGoon M.D., McLaughlin V.V., Roecker E.B. (2008). Ambrisentan for the treatment of pulmonary arterial hypertension: Results of the ambrisentan in pulmonary arterial hypertension, randomized, double-Blind, placebo-controlled, multicenter, efficacy (ARIES) study 1 and 2. Circulation.

[57] Fischer A., Denton C.P., Matucci-Cerinic M., Gillies H., Blair C., Tislow J., Nathan S.D. (2016). Ambrisentan response in connective tissue disease-associated pulmonary arterial hypertension (CTD-PAH)—A subgroup analysis of the ARIES-E clinical trial. Respir. Med..

[58] Rubin L.J., Badesch D.B., Barst R.J., Galiè N., Black C.M., Keogh A., Pulido T., Frost A., Roux S., Leconte I. (2002). Bosentan therapy for pulmonary arterial hypertension. N. Engl. J. Med..

[59] Matucci-Cerinic M., Denton C.P., Furst D.E., Mayes M.D., Hsu V.M., Carpentier P., Wigley F.M., Black C.M., Fessler B.J., Merke P.A. (2011). Bosentan treatment of digital ulcers related to systemic sclerosis: Results from the RAPIDS-2 randomised, double-blind, placebo-controlled trial. Ann. Rheum. Dis..

[60] Mercurio V., Bianco A., Campi G., Cuomo A., Diab N., Mancini A., Parrella P., Petretta M., Hassoun P.M., Bonaduce D. (2018). New Drugs, Therapeutic Strategies, and Future Direction for the Treatment of Pulmonary Arterial Hypertension. Curr. Med. Chem..

[61] Pulido T., Adzerikho I., Channick R.N., Delcroix M., Galiè N., Ghofrani H.-A., Jansa P., Jing Z.-C., Le Brun F.-O., Mehta S. (2013). Macitentan and Morbidity and Mortality in Pulmonary Arterial Hypertension. N. Engl. J. Med..

[62] Rhee R.L., Gabler N.B., Sangani S., Praestgaard A., Merkel P.A., Kawut S.M. (2015). Comparison of treatment response in idiopathic and connective tissue disease-associated pulmonary arterial hypertension. Am. J. Respir. Crit. Care Med..

[63] Chin K., Kim N., Channick R., Muros-Le Rouzic E., Selej M., McLaughlin V. (2017). OPUS Registry: Treatment Patterns and Safety of Macitentan in Patients With Pulmonary Arterial Hypertension Associated With Systemic Sclerosis (PAH-SSc). Chest.

[64] McLaughlin V., Channick R., Kim N., Lammi M., Sulica R., Brand M., Flynn M., Leroy S., Morganti A., Chin K. (2019). Macitentan in Pulmonary Arterial Hypertension Associated With Connective Tissue Disease: Real-World Evidence From the Combined Opus/Orpheus Data Sets. Chest.

[65] Galiè N., Müller K., Scalise A.V., Grünig E. (2015). Patent Plus: A blinded, randomised and extension study of riociguat plus sildenafil in pulmonary arterial hypertension. Eur. Respir. J..

[66] Galiè N., Ghofrani H.A., Torbicki A., Barst R.J., Rubin L.J., Badesch D., Fleming T., Parpia T., Burgess G., Branzi A. (2005). Sildenafil citrate therapy for pulmonary arterial hypertension. N. Engl. J. Med..

[67] Badesch D.B., Hill N.S., Burgess G., Rubin L.J., Barst R.J., Galiè N., Simonneau G. (2007). Sildenafil for pulmonary arterial hypertension associated with connective tissue disease. J. Rheumatol..

[68] Galiè N., Brundage B.H., Ghofrani H.A., Oudiz R.J., Simonneau G., Safdar Z., Shapiro S., White R.J., Chan M., Beardsworth A. (2009). Tadalafil therapy for pulmonary arterial hypertension. Circulation.

[69] Goudie A.R., Lipworth B.J., Hopkinson P.J., Wei L., Struthers A.D. (2014). Tadalafil in patients with chronic obstructive pulmonary disease: A randomised, double-blind, parallel-group, placebo-controlled trial. Lancet Respir. Med..

[70] Kowal-Bielecka O., Fransen J., Avouac J., Becker M., Kulak A., Allanore Y., Distler O., Clements P., Cutolo M., Czirjak L. (2017). Update of EULAR recommendations for the treatment of systemic sclerosis. Ann. Rheum. Dis..

[71] Ghofrani H.A., Galiè N., Grimminger F., Grünig E., Humbert M., Jing Z.C., Keogh A.M., Langleben D., Kilama M.O., Fritsch A. (2013). Riociguat for the treatment of pulmonary arterial hypertension. N. Engl. J. Med..

[72] Rubin L.J., Galié N., Grimminger F., Grünig E., Humbert M., Jing Z.C., Keogh A., Langleben D., Fritsch A., Menezes F. (2015). Riociguat for the treatment of pulmonary arterial hypertension: A long-term extension study (patent-2). Eur. Respir. J..

[73] Humbert M., Coghlan J.G., Ghofrani H.A., Grimminger F., He J.G., Riemekasten G., Vizza C.D., Boeckenhoff A., Meier C., De Oliveira Pena J. (2017). Riociguat for the treatment of pulmonary arterial hypertension associated with connective tissue disease: Results from PATENT-1 and PATENT-2. Ann. Rheum. Dis..

[74] Sitbon O., Channick R., Chin K.M., Frey A., Gaine S., Galiè N., Ghofrani H.A., Hoeper M.M., Lang I.M., Preiss R. (2015). Selexipag for the treatment of pulmonary arterial hypertension. N. Engl. J. Med..

[75] Gaine S., Chin K., Coghlan G., Channick R., Di Scala L., Galiè N., Ghofrani H.A., Lang I.M., McLaughlin V., Preiss R. (2017). Selexipag for the treatment of connective tissue disease-associated pulmonary arterial hypertension. Eur. Respir. J..

[76] Klings E.S., Hill N.S., Ieong M.H., Simms R.W., Korn J.H., Farber H.W. (1999). Systemic sclerosis-associated pulmonary hypertension: Short- and long- term effects of epoprostenol (prostacyclin). Arthritis Rheum..

[77] Oudiz R.J., Schilz R.J., Barst R.J., Galié N., Rich S., Rubin L.J., Simonneau G. (2004). Treprostinil, a prostacyclin analogue, in pulmonary arterial hypertension associated with connective tissue disease. Chest.

[78] Caravita S., Wu S.C., Secchi M.B., Dadone V., Bencini C., Pierini S. (2011). Long-term effects of intermittent Iloprost infusion on pulmonary arterial pressure in connective tissue disease. Eur. J. Intern. Med..

[79] Galiè N., Channick R.N., Frantz R.P., Grünig E., Jing Z.C., Moiseeva O., Preston I.R., Pulido T., Safdar Z., Tamura Y. (2019). Risk stratification and medical therapy of pulmonary arterial hypertension. Eur. Respir. J..

[80] Benza R.L., Miller D.P., Gomberg-Maitland M., Frantz R.P., Foreman A.J., Coffey C.S., Frost A., Barst R.J., Badesch D.B., Elliott C.G. (2010). Predicting survival in pulmonary arterial hypertension: Insights from the registry to evaluate early and long-term pulmonary arterial hypertension disease management (REVEAL). Circulation.

[81] Boucly A., Weatherald J., Savale L., Jaïs X., Cottin V., Prevot G., Picard F., De Groote P., Jevnikar M., Bergot E. (2017). Risk assessment, prognosis and guideline implementation in pulmonary arterial hypertension. Eur. Respir. J..

[82] Kylhammar D., Kjellström B., Hjalmarsson C., Jansson K., Nisell M., Söderberg S., Wikström G., Rådegran G. (2018). A comprehensive risk stratification at early follow-up determines prognosis in pulmonary arterial hypertension. Eur. Heart J..

[83] Hoeper M.M., Kramer T., Pan Z., Eichstaedt C.A., Spiesshoefer J., Benjamin N., Olsson K.M., Meyer K., Vizza C.D., Vonk-Noordegraaf A. (2017). Mortality in pulmonary arterial hypertension: Prediction by the 2015 European pulmonary hypertension guidelines risk stratification model. Eur. Respir. J..

[84] Mercurio V., Diab N., Peloquin G., Housten-Harris T., Damico R., Kolb T.M., Mathai S.C., Hassoun P.M. (2018). Risk assessment in scleroderma patients with newly diagnosed pulmonary arterial hypertension: Application of the ESC/ERS risk prediction model. Eur. Respir. J..

[85] Gali N., Palazzini M., Manes A. (2010). Pulmonary arterial hypertension: From the kingdom of the near-dead to multiple clinical trial meta-analyses. Eur. Heart J..

[86] Jansa P., Pulido T. (2018). Macitentan in Pulmonary Arterial Hypertension: A Focus on Combination Therapy in the seraphin trial. Am. J. Cardiovasc. Drugs.

[87] Coghlan J.G., Channick R., Chin K., Di Scala L., Galiè N., Ghofrani H.A., Hoeper M.M., Lang I.M., McLaughlin V., Preiss R. (2018). Targeting the Prostacyclin Pathway with Selexipag in Patients with Pulmonary Arterial Hypertension Receiving Double Combination Therapy: Insights from the Randomized Controlled GRIPHON Study. Am. J. Cardiovasc. Drugs.

[88] Galie N., Barbera J.A., Frost A.E., Ghofrani H.A., Hoeper M.M., McLaughlin V.V., Peacock A.J., Simonneau G., Vachiery J.L., Grunig E. (2015). Initial use of ambrisentan plus tadalafil in pulmonary arterial hypertension. N. Engl. J. Med..

[89] Coghlan J.G., Galiè N., Barberà J.A., Frost A.E., Ghofrani H.A., Hoeper M.M., Kuwana M., McLaughlin V.V., Peacock A.J., Simonneau G. (2017). Initial combination therapy with ambrisentan and tadalafil in connective tissue disease-associated pulmonary arterial hypertension (CTD-PAH): Subgroup analysis from the AMBITION trial. Ann. Rheum. Dis..

[90] Hassoun P.M., Zamanian R.T., Damico R., Lechtzin N., Khair R., Kolb T.M., Tedford R.J., Hulme O.L., Housten T., Pisanello C. (2015). Ambrisentan and tadalafil up-front combination therapy in scleroderma-associated pulmonary arterial hypertension. Am. J. Respir. Crit. Care Med..

[91] (2016). NCT02682511 Oral Ifetroban to Treat Diffuse Cutaneous Systemic Sclerosis (SSc) or SSc-associated Pulmonary Arterial Hypertension. NCT02682511.

[92] (2016). NCT02981082 Dimethyl Fumarate (DMF) in Systemic Sclerosis-Associated Pulmonary Arterial Hypertension. NCT02981082.

[93] EUCTR2016-004793-17-DE Bardoxolone Methyl Evaluation in Patients with Pulmonary Hypertension (PH). http://www.who.int/trialsearch/Trial2.aspx?TrialID=EUCTR2016-004793-17-DE.

[94] (2010). NCT01086540 Rituximab for Treatment of Systemic Sclerosis-Associated Pulmonary Arterial Hypertension (SSc-PAH). NCT01086540.

[95] Zamanian R., Badesch D., Chung L., Domsic R., Medsger T., Pinckney A., Keyes-Elstein L., D’Aveta C., Spychala M., White J. (2019). Late Breaking Abstract—Safety and efficacy of B-cell depletion with rituximab for the treatment of systemic sclerosis-associated pulmonary arterial hypertension. Eur. Respir. J..

